# Design, fabrication, commissioning, and dosimetric verification of a GRID collimator for proton SFRT on a compact proton therapy machine

**DOI:** 10.1002/mp.17939

**Published:** 2025-07-15

**Authors:** Jufri Setianegara, Winter Green, Xiandong Zhao, Thomas R. Mazur, Arash Darafsheh, Anthony J. Apicelli, Shahed N. Badiyan, Stephanie M. Perkins, Tianyu Zhao, Michael T. Prusator

**Affiliations:** ^1^ Department of Radiation Oncology University of Pennsylvania Philadelphia Pennsylvania USA; ^2^ Department of Radiation Oncology Washington University School of Medicine St. Louis Missouri USA; ^3^ Department of Radiation Oncology University of Texas Southwestern Medical Center Dallas Texas USA; ^4^ Department of Radiation Oncology University of South Florida Tampa Florida USA

**Keywords:** collimator, GRID radiotherapy, proton therapy, spatially fractionated radiation therapy, synchrocyclotron

## Abstract

**Background:**

Proton GRID radiotherapy (RT) is an extension of photon spatially fractionated radiation therapy (SFRT) techniques for bulky invasive tumors. It has been hypothesized that GRID RT improves the therapeutic ratio by minimizing normal tissue toxicities associated with treating bulky volumes while inducing abscopal radiobiologic effects. However, compact, synchrocyclotron‐based proton therapy machines with large spot sizes pose unique technical challenges in implementing proton GRID RT.

**Purpose:**

The purpose of this work is to (a) design and model a collimating brass aperture within the RayStation treatment planning system (TPS), (b) validate the designed aperture by creating a commissioning plan and measuring the absolute and relative proton dose distributions delivered, and (c) perform a robustness analysis to determine the allowable mechanical tolerances and uncertainties during treatment delivery.

**Methods:**

A custom (27 × 21.5 × 5 cm^3^) brass collimator (.decimal) was designed and fabricated with divergently‐matched circular holes of 15‐mm‐diameter arranged in a hexagonal pattern. In‐house RayStation scripts were developed to import the computer‐aided design (CAD) model of the collimator into the TPS, and accurately orient and position the collimator as “support structures” for each beam angle requiring the collimator on a given plan. Divergently‐matched cylindrical optimization structures were then created with 5, 10 and 15 mm diameters. Commissioning plans were created to deliver uniform proton physical doses (50 cGy and 8 Gy) through each aperture to 5–15 cm depth within a water phantom. One dimensional (1D) and 2D proton dose measurements were performed with various available radiation detectors, including: (a) PPC05 parallel‐plate ion chamber, (b) MatriXX ion chamber array, (c) Lynx scintillation detector, and (d) Gafchromic EBT3 radiochromic films. Gamma analyses at 3%/3 mm criteria were performed for the 2D dose measurements acquired with the MatriXX and Lynx detectors. Finally, mounting errors were simulated within the TPS by artificially displacing the brass aperture along the crossline, inline and snout extension directions to determine the minimum allowable mechanical deviations between the TPS and the actual mounted aperture position.

**Results:**

Experimental measurements showed the best dosimetric agreements with the TPS calculations for optimization cylinder diameters of 10 mm with gamma passing rates of at least 97.9%. 1D absolute proton dose measurements with an ionization chamber showed agreement within 2.11% of TPS calculations once correcting for partial‐volume averaging. Simulated mounting or setup errors within the TPS indicated a lateral positional requirement of ± 1.5 mm and a longitudinal snout positional requirement of ± 3 cm to achieve gamma passing rates of at least 90% (institutional standards).

**Conclusion:**

We have commissioned a brass collimator consisting of milled divergent apertures for clinical SFRT treatments via a proton GRID technique. This process included an assessment of dosimetric sensitivity of aperture positioning error, and also dosimetric evaluation of the aperture model as a brass support structure within the TPS. Future works entail the creation of clinical SFRT plans using different planning techniques and their respective dose comparisons.

## 1 INTRODUCTION

Spatially fractionated radiation therapy (SFRT) was originally introduced to minimize skin toxicities that were associated with the treatments of deep‐seated tumors using orthovoltage techniques.^1^, ^2^ However, the increased usage of modern megavoltage linear accelerators were more efficacious in skin sparing and SFRT techniques were disregarded in favor of the current paradigm of homogenous tumor dosing. SFRT treatments have since regained clinical interest in the treatment of bulky tumors as demonstrated by Mohiuddin et al. who observed increased safety and efficacy relative to conventional treatment planning techniques.^3^, ^4^, ^5^ Prior to the use of SFRT, dose escalation to debulk these large tumors could not be safely performed due to the inability of patients to tolerate the severe normal tissue toxicities that are typically associated with the treatment of bulky tumor volumes.^6^ Treatment planning utilizing SFRT approaches involves creating high peak‐to‐valley dose ratios within the bulky tumors which greatly reduces the incidence of grade 3–4 radiotoxicities due to the reduction of the volume of normal tissues receiving a high radiation dose.^7^ Despite the spatial heterogeneity of the tumor coverage, excellent treatment goal efficacies such as local control,^1^, ^8^ and pain and mass effect management were also achieved.^7^ While the underlying radiobiology of SFRT treatment modalities is currently not well‐understood,^9^ there are multiple promising hypotheses—such as leveraging normal cells’ superior repair capabilities,^10^, ^11^, ^12^ bystander and abscopal effects,^13^ tumor microvascular changes,^14^ and radiation‐induced immune‐response^6^, ^9^ – that are the subject of active and ongoing academic and clinical research.^15^, ^16^

Traditionally, SFRT is delivered using a single unopposed field delivering a dose of 10–15 Gy with the spatial modulation created through the use of a hexagonal pattern of circular apertures milled within commercially‐available Cerrobend or brass blocks.^1^, ^2^, ^3^ These 2D planning techniques are commonly referred to within the literature as “GRID” treatments.^15^, ^16^ Since then, technological advances in beam delivery and planning such as the use of multileaf collimators (MLCs) and intensity modulated radiation therapy (IMRT) techniques have allowed for more sophisticated 3D SFRT plans to be created.^17^ This modern 3D SFRT treatment modality is known as “LATTICE” radiation therapy (LRT) which achieves spatial modulation through the creation of high dose vertices in the form of spheres rather than cylinders.^18^ As compared with their historic GRID counterparts, LRT can achieve a highly customizable spatial and dosimetric distribution of these high dose regions within the bulky tumor along with better controlled organ‐at‐risk (OAR) sparing. Modern LRT treatments have been successfully implemented and are the subject of multiple safety and clinical efficacy studies.^19^, ^20^, ^21^, ^22^

While the bulk of SFRT treatments have been delivered using photon beams, there has been growing clinical interests in creating SFRT dose distributions using proton beams.^23^, ^24^ This is because proton beams have a finite range in medium and deposit the bulk of their dose within the Bragg peak.^25^ This finite range can confer superior distal sparing and can be clinically advantageous in the retreatment setting, and in treating pediatric patients or patients with tumors that are closely abutting critical serial OARs.^26^ However, the creation of clinical proton SFRT plans is challenging, especially if lower energy protons are required to treat superficial bulky tumors. This is because lower energy protons tend to experience elastic multiple Coulomb scattering (MCS) both in air and within the patient^25^ which will result in increased proton spot sizes^27^, ^28^, ^29^ that are non‐ideal for SFRT plans. This undesirable effect is worsened for isochronous cyclotrons and synchrocyclotrons as physical range shifters are used within the fixed‐energy proton beamline to degrade the proton energies which will also result in further MCS interactions.^30^, ^31^ While this effect can be mitigated through the use of an energy selection system (ESS) which incorporates quadrupole focusing magnets to achromatically re‐focus the proton spot, the ESS is absent for the most single room compact proton machine designs^32^ and it may not lead to a proton spot size which is sufficiently small for proton SFRT planning.^31^ There have been various proposed collimating solutions to trim the lateral penumbra of these lower energy proton spots such as dynamic collimator systems (DCS) and multi‐leaf collimators (MLCs)^31^, ^32^, ^33^ but current commercially available collimator systems^32^ are not of a sufficient spatial resolution to alleviate the challenges of delivering clinical proton SFRT plans.

In this study, we demonstrate (i) the feasibility of designing physical collimating brass apertures for lateral penumbra spreading, (ii) implementing them successfully within a commercial proton TPS to be used for proton SFRT treatment planning, and (iii) treatment delivery from a compact proton therapy machine.

## 2 METHODS AND MATERIALS

### 2.1 Mevion S250i hyperscan beamline

The Mevion S250i Hyperscan (Mevion Medical Systems, Littleton, MA) system is a single‐room gantry‐mounted compact proton synchrocyclotron with proton pencil beam scanning (PBS) capability that is enabled by a dual axis scanning magnet in the beamline.^28^ Proton beams at a fixed energy of 227 MeV (∼32 cm range in water) are extracted from the 8.5 T Nb_3_Sn superconducting synchrocyclotron while energy modulation is achieved within the proton gantry nozzle using a fast energy modulation system (EMS) consisting of 18 Lexan (1.20 g/cm^3^) plates of different thicknesses to create 161 different proton energies with a minimum step size of 2.1 mm in water‐equivalent‐thickness (WET) and with an energy switching time of approximately 50 ms.^28^ Lateral penumbra can be trimmed using an adaptive aperture (AA) module which is also located within the proton gantry nozzle. The AA sharpens the lateral penumbra using the principles of DCS^34^ and its module consists of five opposing pairs of 5‐mm‐thick nickel‐alloy leaves which are located on two opposing leaf carriages. Located above and below these 5‐mm‐thick leaves are an opposing pair of thicker 20 mm jaws.^32^ The AA module can be used to trim the proton spots per energy layer with a maximum speed of 1.2 s per spot.^28^ During beam delivery, the spot position, shape, and charge of the proton beam is continuously monitored by a dose delivery system (DDS) consisting of six transmission ionization chambers: (a) two wide integral ionization chambers and (b) two pairs of strip chambers each orthogonal to each other.

### 2.2 Proposed workflow for proton GRID clinical treatments at WashU

**FIGURE 1:**
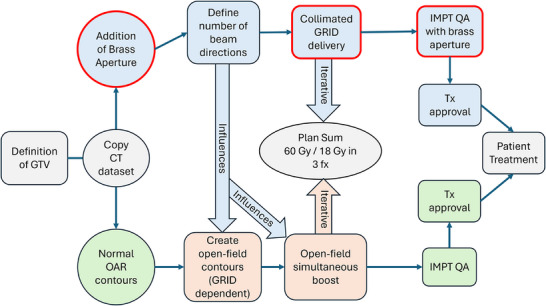
Proposed clinical workflow for clinical GRID treatments at our institution. The proposed GRID SFRT prescription will be 18 Gy (RBE) to the entire GTV while creating 60 Gy (RBE) vertices within 5 mm of the GTV in simultaneously 3 fractions. The GRID SFRT treatment plan will be separately optimized in 2 parts: (1) with (blue arm) and (2) without (green arm) the brass aperture. The number of GRID fields chosen for the treatment plan will influence the planning technique for the treatment plan without the brass aperture (orange bubble). These works will impact the manner in which the GRID SFRT plans are created along with their accompanying patient‐specific QA procedures (denoted by red outlines).

Figure 1 illustrates the proposed clinical workflow for GRID proton treatments within our institution. While out of the scope of this work, we intend to treat patients with unresectable or metastatic lesions palliatively with a prescription of 60 Gy (RBE) and 18 Gy (RBE) simultaneously in three fractions, that is the entire GTV covered with 18 Gy (RBE) while creating 60 Gy (RBE) vertices within 5 mm of the GTV. We plan to deliver this GRID SFRT treatment in two parts: (1) with the brass aperture as denoted by the blue arm in Figure 1 and without the brass aperture as denoted by the green arm and otherwise known as “open field”. Although these plans will be optimized separately on two different CT datasets, the GRID planning technique will influence the open field planning technique as denoted by the orange parts in Figure 1. The current work is required for the accurate inclusion of the brass aperture within the CT dataset, along with proper treatment planning techniques and patient‐specific QA measurements as denoted in the red contour.

The choice of the 15 mm milled apertures in a hexagonal pattern was inspired by our current clinical experience in photon lattice SBRT.^19^, ^20^ The other physical aspects of the collimator such as its thickness, length, breadth and divergence are straightforward and designed to cover the entire aspect of the proton field, being thick enough to attenuate the highest energy protons and being divergent‐matched to our machine's beam focal point.

### 2.3 Development of collimating brass aperture

**FIGURE 2:**
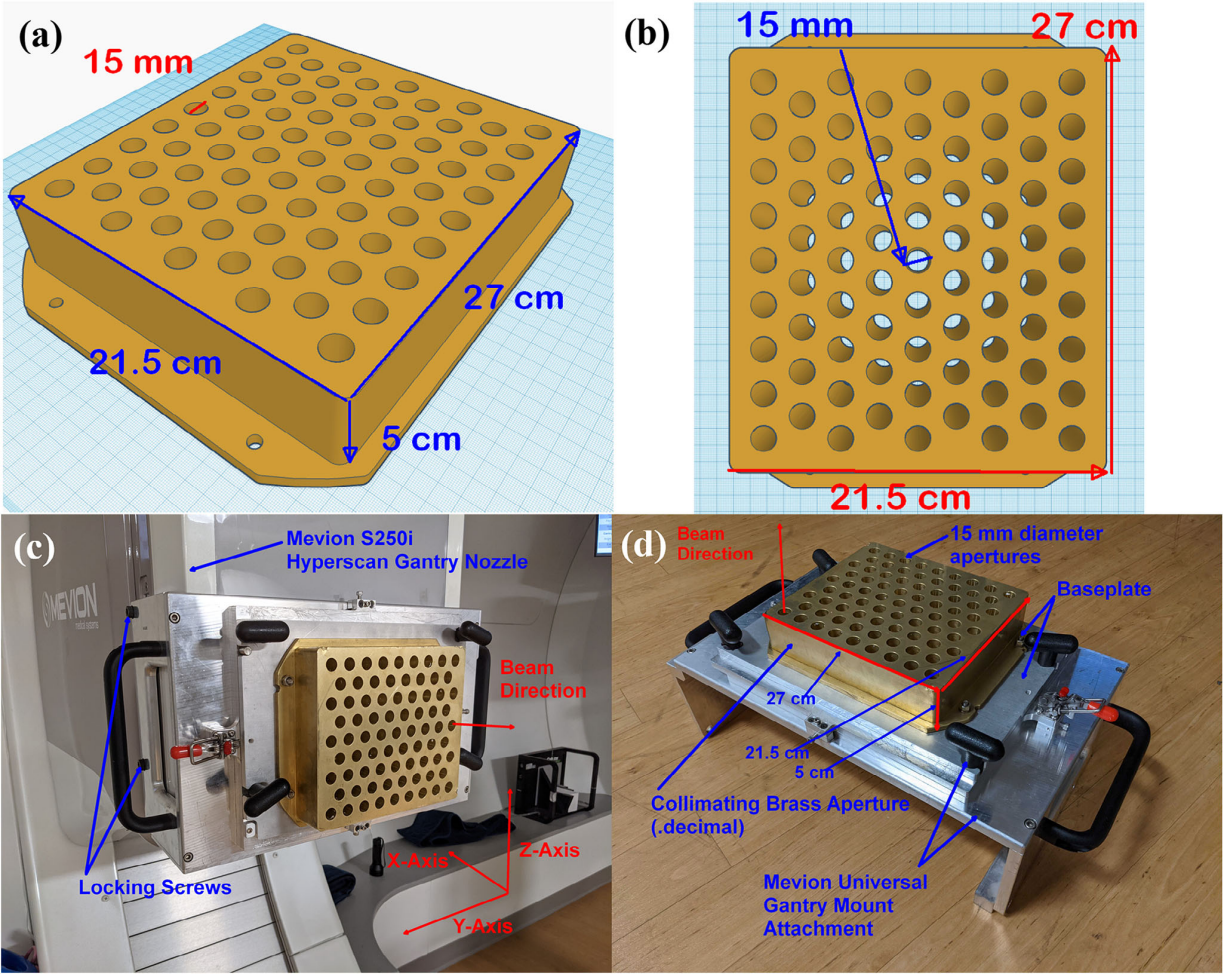
(a,b) Schematic of the collimator brass aperture design within Tinkercad. (c,d): Photographs of collimating brass aperture (.decimal) of dimensions 27 × 21.5 × 5 cm^3^ mounted upon a (c): Mevion universal gantry nozzle mount attachment and (d): upon the entrance window of the gantry nozzle at a gantry angle of 90°. 15 mm diameter holes are machined throughout the collimating brass aperture. Beam divergence was accounted for by angling each hole to match the divergence angle while keeping the hole diameter constant. The isocenters of the treatment room are labelled. Please note that the orientation of the brass apertures’ physical axes with respect to the treatment room's isocenter axes are gantry angle dependent.

A custom 27 × 21.5 × 5 cm^3^ brass collimator (.decimal, Sanford, FL) weighing approximately 16 kg was designed and created with a divergently matched hexagonal pattern of circular apertures of 15 mm diameter milled throughout the brass collimator (Figure 2). A total of 77 such circular apertures are milled and arranged in 9 rows. The number of circular apertures within each row alternates between 8 and 9 to generate the hexagonal pattern. The brass collimator was mounted on a steel baseplate using 4 hex screws (Figure 2c). The steel baseplate is mounted securely upon the Mevion universal gantry nozzle mount attachment using four additional larger screws (Figure 2c). Finally, the entire mount attachment was mounted over the entrance window of the gantry nozzle using four longer hex screws (Figure 2d). When mounted on the gantry nozzle, the brass aperture was found to fit snugly and reproducibly with no discernable play in its position relative to the entrance window. The brass collimator was designed to be divergence‐matched to the proton beam at a transverse plane that is 19.5 cm from the beam's isocenter. The collimator was positioned correctly with a snout extension of 23.5 cm which accounted for a systematic 4 cm offset between the snout and the universal mount.

### 2.4 Modeling collimating brass aperture within TPS

A 3D computer aided design (CAD) model of the collimating brass aperture was created in the form of a stereolithography (.stl) file and was used by the manufacturer (.decimal) for its physical construction. A contour representation of the brass aperture was created within the RayStation (Version 11a, RaySearch Laboratories, Stockholm, Sweden) TPS by reading the .stl file and importing the CAD model within the “Patient Modeling” workspace using the TPS’ scripting environment. The imported .stl contour was then positioned correctly over all of the gantry angles through the use of 4 × 4 transformation matrices within the TPS (Equation 1) where R_11_ to R_33_, correspond to a standard rotational and scaling transformation matrix about the *x*, *y* and *z* coordinates with the remaining elements corresponding to shear transformations that were kept at unity.

(1) $$\;R_{\mathrm{xy}}=\left(\begin{array}{cccc} R_{11} & R_{12} & R_{13} & R_{14}\\ R_{21} & R_{22} & R_{23} & R_{24}\\ R_{31} & R_{32} & R_{33} & R_{34}\\ R_{41} & R_{42} & R_{43} & R_{44} \end{array}\right)\;$$

With this transformation matrix, the collimator was (1) brought to isocenter, (2) re‐oriented in the +z direction, (3) offset by 4 cm to account for the physical separation between the snout and the mounting system, and (4) positioned over all gantry angles relative to the isocenter. Step (4) depends on the gantry and couch angles $\theta_{g}$ and $\theta_{c}$ as seen in Equation 2.

(2) $$\;R_{\mathrm{xy}}^{\mathrm{beam}}=\left(\begin{array}{cccc} \cos\theta_{g}\cos\theta_{c} & -\sin\theta_{g}\cos\theta_{c} & \sin\theta_{c} & B_{x}\\ \sin\theta_{g} & \cos\theta_{g} & 0 & B_{y}\\ -\cos\theta_{g}\sin\theta_{c} & \sin\theta_{g}\sin\theta_{c} & \cos\theta_{c} & B_{z}\\ 0 & 0 & 0 & 1 \end{array}\right)\;$$

**FIGURE 3:**
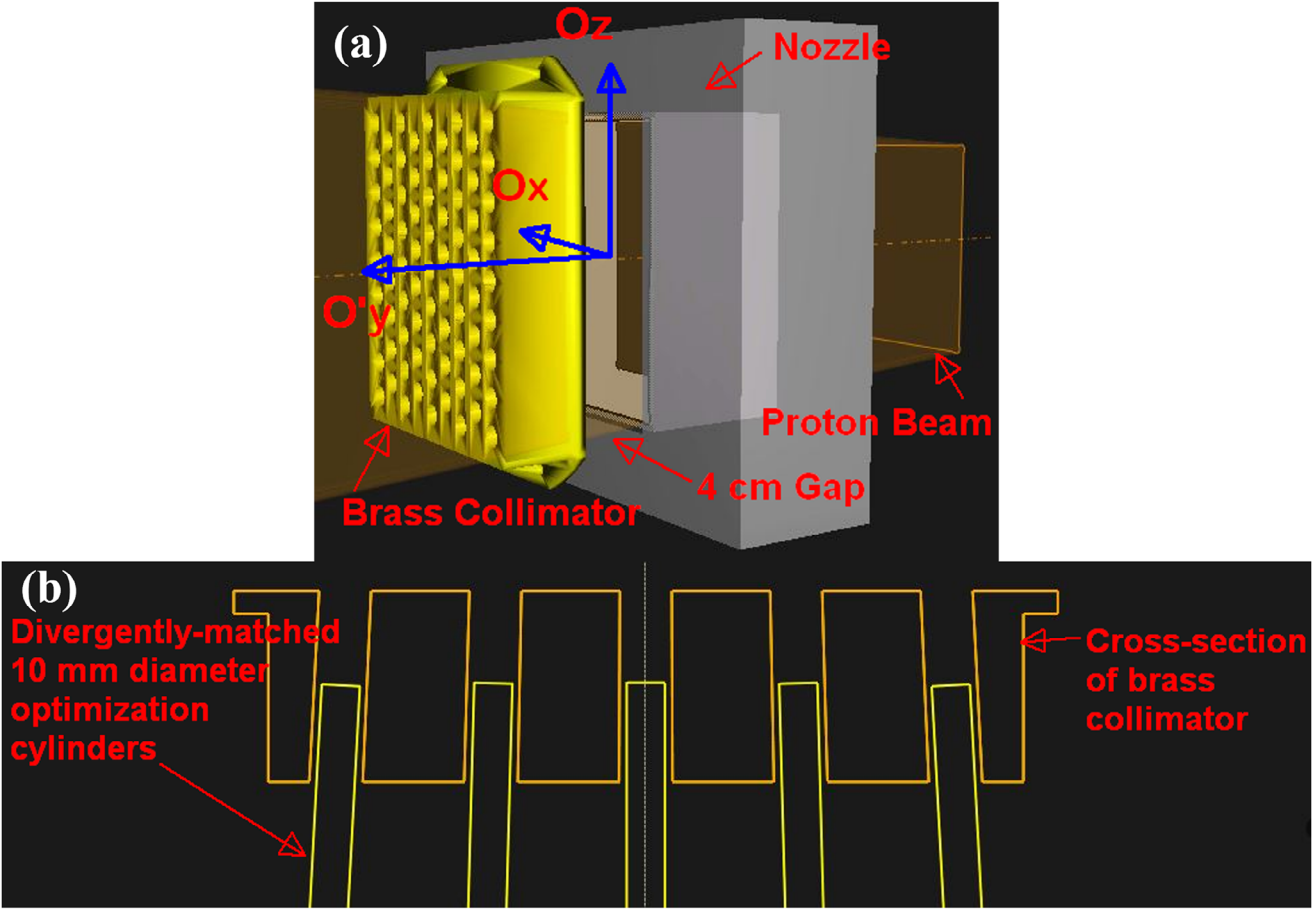
Screenshots of brass collimator within the RayStation TPS. (a): Initial positioning and orientation of the brass collimator with respect to a gantry angle of 180°. $O_{x,y,z}$ corresponds to the initial beam's isocenter coordinates and $O_{y}^{\prime }$ in Equation 1 refers to the shifted isocenter coordinates to account for the 4 cm gap between the snout and the brass aperture as illustrated. (b): 2D cross‐section view of these fitted optimization cylinders. Care was undertaken to ensure that the cylinders are well‐aligned along the milled aperture channels of the brass collimator. TPS, treatment planning system.

For ease of treatment planning, cylindrical optimization structures were fitted over all apertures as seen in Figure 3. When fitted correctly and accurately, these optimization cylinders reveal the intersections of the openings of the brass aperture with the PTV to generate optimization targets that can be used for SFRT treatment planning.

(3) $$\;R_{\mathrm{xy}}^{\mathrm{cyl}}=\left(\begin{array}{cccc} \cos\theta_{x} & -\sin\theta_{x} & 0 & C_{x}\\ -\sin\theta_{x}\cos\theta_{y} & \cos\theta_{x}\cos\theta_{y} & -\sin\theta_{y} & C_{y}\\ \sin\theta_{x}\sin\theta_{y} & \cos\theta_{x}\sin\theta_{y} & \cos\theta_{y} & C_{z}\\ 0 & 0 & 0 & 1 \end{array}\right)\;$$

These cylinders were systematically translated by the nominal hexagonal center‐to‐center distances (*x*: 2.035 cm, *y*: 2.350 cm) and rotated by the nominal angular divergences ($\theta_{x}$ = 0.375°/cm, $\theta_{y}$ = 0.324°/cm) of the brass aperture's design (Equation 3). Cylinder diameters of 5, 10, and 15 mm were created to test for optimal cylinder diameter for proton GRID treatment planning.

Finally, the brass aperture's material is set to “Brass” within the “ROI properties” of the contour with a mass density of 8.4 g/cm^3^ and its region‐of‐interest (ROI) type set to “Support” structure. The plan's dose grid was expanded to include this structure for dose calculations.

### 2.5 Creation of validation proton GRID plan

**FIGURE 4:**
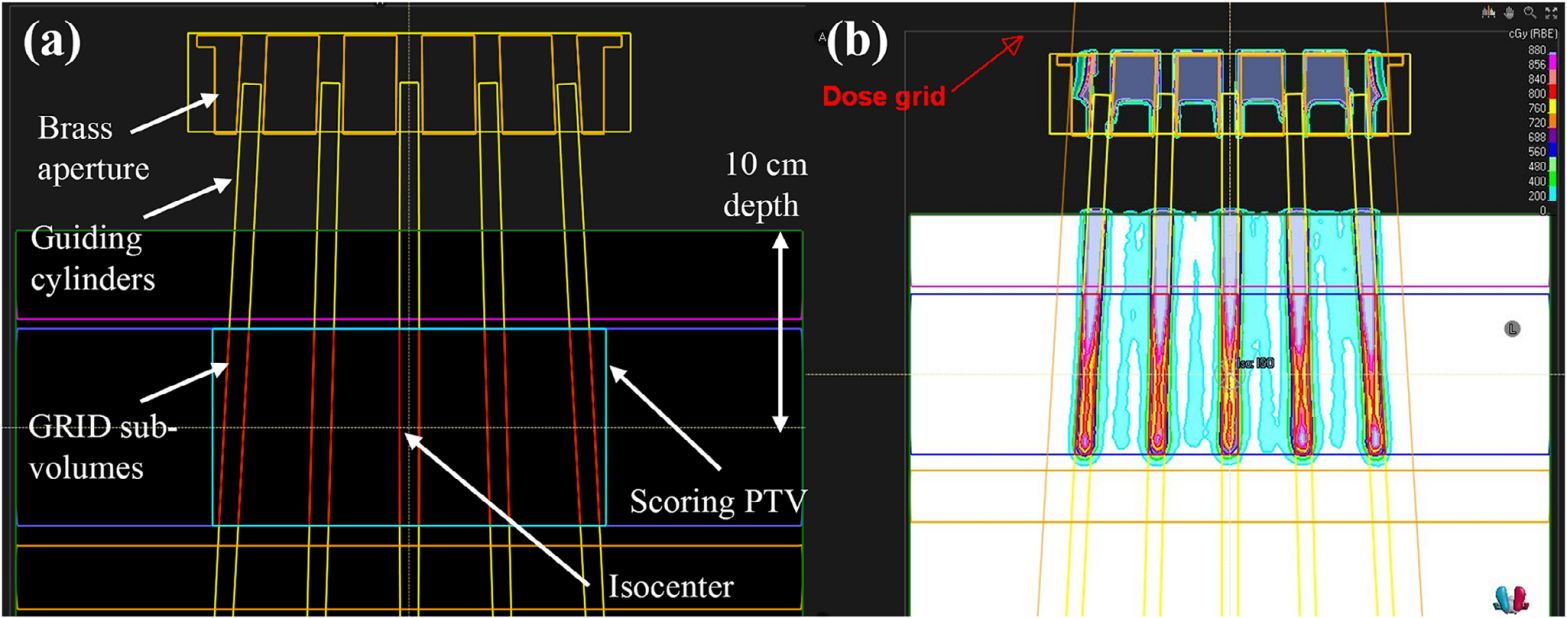
Verification proton GRID plans were created to assess the accuracy of the placement of the 77 optimization cylinders namely (a): optimization structures and (b): dose distributions. An approximate “Scoring PTV” volume was constructed between depths of 5–15 cm which encompassed all GRID sub‐volumes that were generated from the guiding cylinders fitted over all holes of the brass aperture.

Figure 4 depicts the proton GRID validation plan that was created with a single anterior‐posterior (AP) beam with an isocenter that is 10 cm deep within the phantom. The cylindrical optimization structures that were previously generated over all holes were made to be cropped between depths of 5 and 15 cm within the virtual phantom. Validation plans (“high‐dose verification plan”) were created for cylinder diameters ranging 5–15 mm with coverage objectives *D*_95%_ > 720 cGy (RBE) to these cropped cylinders. The depths and dose levels were justified as they represented the approximate clinical conditions for which this technique would be used for institutionally (palliative bulky sarcomas). In addition, we also created verification plans that optimizes for a lower dose coverage of *D*_95%_ > 45 cGy (RBE) (“low‐dose verification plan”) to be delivered to measurement devices with a lower dynamic range to prevent detector saturations.

Dose reporting was performed in accordance with the recommendations that were provided by Zhang et al.^16^ An approximate bulky PTV scoring volume was constructed by creating a 23 x 20 x 10 cm cuboid volume (cyan contour of Figure 4) that encompassed all high‐dose cropped cylinders (red contours of Figure 4) with an approximate margin of 0.5 cm which represents a safety margin that would be used for clinical GRID planning. As per the recommendations,^16^ the *D*_90_, *D*_50_, *D*_20_, *D*_10_, *D*_5_, *D*_mean_, peak‐to‐valley‐dose‐ratio (PVDR), *D*_90_/*D*_10_ and equivalent‐uniform‐dose (EUD) were reported.

### 2.6 1D and 2D dose measurements of proton GRID plan

**FIGURE 5:**
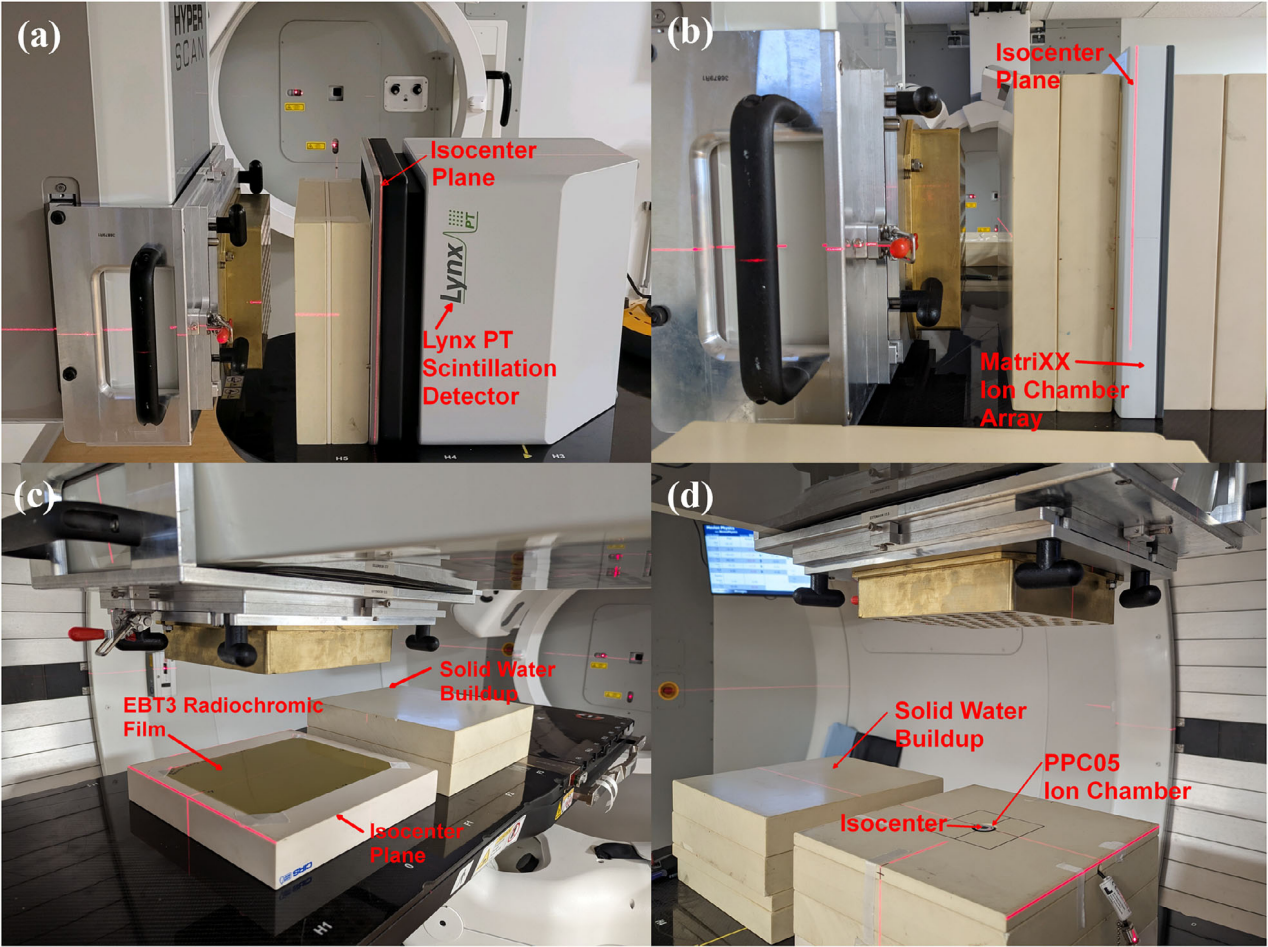
Experimental setups for 2D and 1D proton dose verification measurements using (a): Lynx PT scintillation detector, (b): MatriXX ionization chamber array, (c): Gafchromic EBT3 radiochromic films and (d): PPC05 plane‐parallel ionization chamber. Low‐dose verification plan was delivered to the Lynx detector with an iris setting of 50 while the high‐dose verification plan was delivered to the rest of the detectors. The sensitive elements of all detectors were aligned along the isocenter plane, and solid water was used to create a measurement depth of 10 cm within water.

2D experimental measurements were performed using a (a) MatriXX PT ionization chamber detector array (IBA Dosimetry Inc.), (b) Lynx PT scintillation detector (IBA Dosimetry Inc.), and (c) Gafchromic EBT3 films (Ashland Advanced Materials Inc.) as seen in Figure 5. The MatriXX PT is made up of 1020 air‐vented ionization chambers in a 32 × 32 arrangement contained within an active measurement area of 24.4 × 24.4 cm^2^ with a pixel spacing of 7.6 mm.^35^ The Lynx includes an inorganic scintillation screen with an active detector area of 30.0 × 30.0 cm^2^ and a pixel spacing of 0.5 mm.^36^ Due to the ease of saturation of the Lynx's charge‐coupled device (CCD) camera, the low‐dose verification plan was delivered with an iris setting of 50. All 2D dose measurements were performed with their respective sensitive elements positioned at the isocenter (SAD setup) at a depth of 10 cm in solid water. Absolute dosimetry was established for the MatriXX prior to experimental measurements by cross‐calibrating it against an ADCL‐calibrated PPC05 ionization chamber (IBA Dosimetry Inc.) under TRS‐398 conditions–227.1 MeV, 5 cm depth, 10 × 10 cm^2^ size, 1681 total spots, 0.25 cm spot spacing, and 1 monitor unit (MU) per spot.^37^ Absolute dosimetry was established for EBT3 film measurements by constructing an initial dose calibration curve (0.25–12 Gy) under standard calibration conditions^29^, ^37^ with 48 h post‐irradiation readouts using an Epson 12000 XL flatbed scanner (Epson Inc.). Calibrations curves consist of a polynomial fit performed over the optical density (OD) of the red channel.^29^, ^37^ 1D experimental dose measurements were also performed with an ADCL‐calibrated PPC05 ionization chamber that is placed at a depth of 10 cm under an SAD setup. Reference doses were obtained from the TPS by (a) contouring a cylindrical volume of 9.9 mm diameter on a single slice at the isocenter, (b) re‐calculating with 1 mm dose resolution and (c) obtaining the average proton dose to this active volume contour to account for partial volume averaging. For our institution's QA policy, an absolute dose agreement within 3% is required prior to treatment.

### 2.7 Gamma analysis

Gamma analysis^38^ was performed using both the manufacturer's software (myQA Patients, IBA Dosimetry) and cross‐verified using an independent open‐source library^39^ (PyMedPhys) for all 2D dose measurements. For Lynx measurements which consisted of relative counts ranging from 0–1000, the maximum measured pixel count was normalized to a relative value of 100%. Gamma criteria of 3 mm distance‐to‐agreement (DTA) and 3% dose difference were applied along with a global low dose threshold of 10%. For our institution's QA policy, a 3%/3 mm passing rate of 90% is required prior to treatment.

### 2.8 Simulation of positional errors

Potential mis‐positioning of the brass collimator was investigated to characterize possible dosimetric impact. These positional errors were simulated by (a) artificially shifting the brass collimator in all 3 axes within the TPS, (b) recalculating the verification plan with these systematic positional shifts and (c) performing a 2D gamma analysis at a gamma criterion of 3%/3 mm with actual experimental measurements (Lynx detector, low‐dose verification plan, 10 mm optimization cylinder diameter) using PyMedPhys.

## 3 RESULTS

### 3.1 Low‐dose verification gamma analysis using MatriXX and Lynx detectors

**FIGURE 6:**
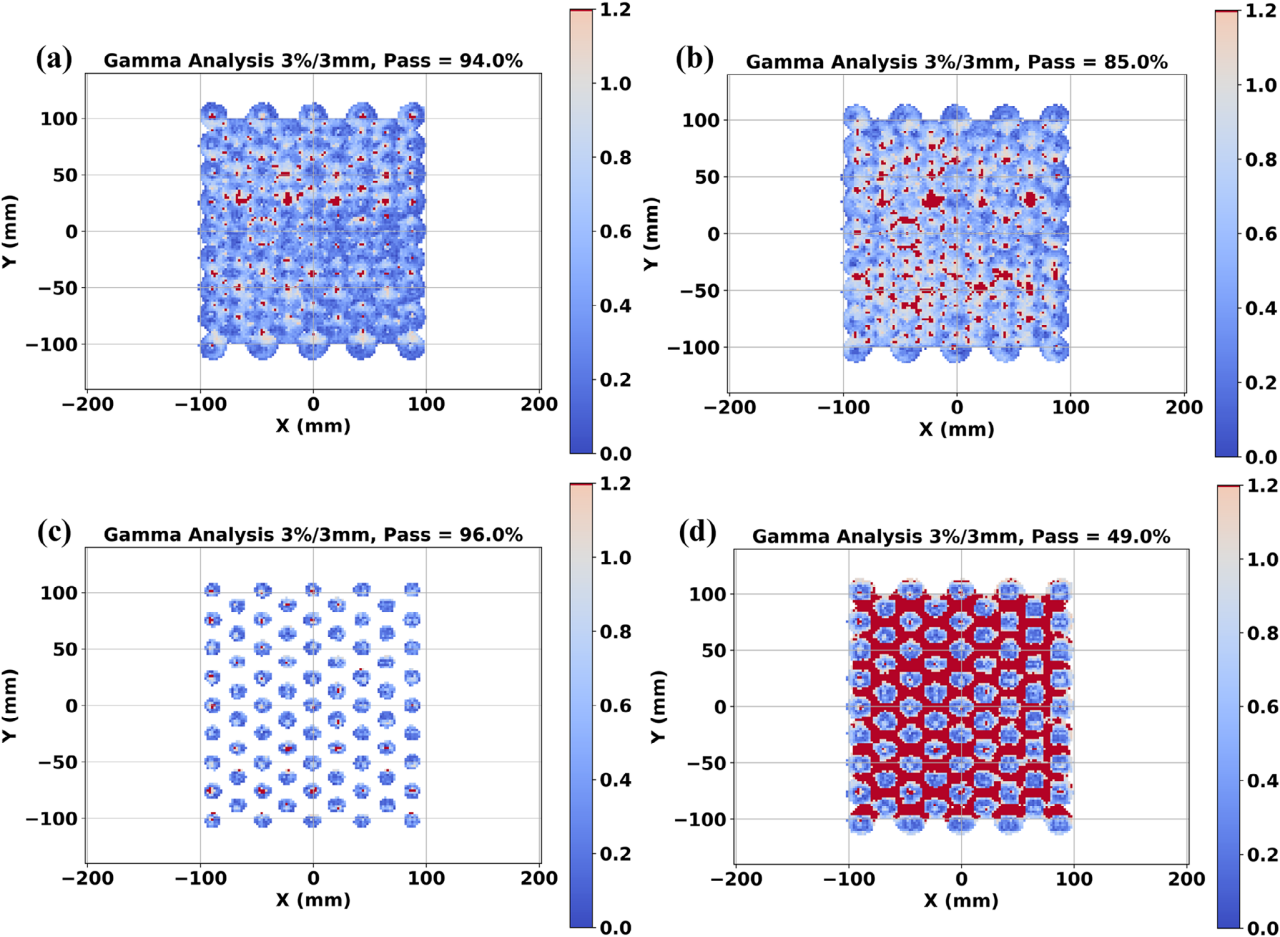
Independent 2D gamma analysis performed using PyMedPhys for (a,b): 2D Lynx (iris setting of 50%) and (c,d): MatriXX measurements. (a): 10 mm cylinder diameters showed a higher gamma score of 94.0% as compared to (b): 15 mm diameters gamma scores of 85.0%. Gamma thresholds of (c): 50% for the MatriXX detector had a 96.0% passing score as compared to (d): 49.0% experienced by the 10% threshold. Gamma analysis was performed with a 3% and 3 mm gamma criteria, as per institution's standards.

Table 1 contains the results of the gamma analysis comparisons between the reference 2D proton dose distributions and experimental measurements performed with both the MatriXX and Lynx detectors for the low‐dose verification plan utilizing optimization cylinders of various diameters. For the MatriXX detector, a 50% threshold was applied to address its lack of adequate spatial resolution. It was determined that for both MatriXX and Lynx measurements that the best agreements occurred with optimization cylinder diameters that are slightly less than that of the size of the actual machined cylinder apertures. Figure 6 shows the 2D gamma analyses as performed with PyMedPhys comparing 2D experimental measurements on the Lynx detector for the (a): 10 mm and (b): 15 mm optimization cylinder diameters. Failing points on the 15 mm optimization cylinder diameters tend to occur in the boundaries around each aperture perimeter.

**TABLE 1:** Results of gamma analysis at a gamma criterion of 3%/3 mm between experimental measurements from the Lynx (50% iris setting) and MatriXX detectors and reference 2D proton dose distributions exported from the TPS.

	Lynx	MatriXX	
Optimization cylinder diameter	10% Threshold	10% Threshold	50% Threshold
5 mm	97.2%	82.8%	98.8%
10 mm	97.9%	82.3%	98.8%
15 mm	88.1%	71.8%	92.4%

*Note*: Gamma analysis was performed using the myQA Patients software which is currently being used for our institution's IMPT patient‐specific QA measurement and analysis. For these measurements, the low‐dose verification plan was delivered. For our institution's QA policy, a 3%/3 mm passing rate of 90% is required prior to treatment.

Abbreviation: TPS, treatment planning system.

Figure 6 shows the corresponding 2D gamma analyses as performed with PyMedPhys comparing 2D experimental measurements on the MatriXX detector for the low‐dose verification plan optimized with the 10 mm optimization cylinder diameter (Table 1 and Figure 6) with dose thresholds of (a): 50% and (b): 10%. Failing points corresponded to the valley doses in between the peak doses.

### 3.2 Absolute dose measurements with PPC05 ion chamber and MatriXX

Table 2 contains the results of the gamma analysis comparisons between the reference 2D proton dose distributions and experimental measurements performed with the MatriXX for both dose verification plans. As mentioned previously, a 50% threshold was included to address its lack of adequate spatial resolution. Also included are absolute proton dose measurements as performed with a PPC05 ionization chamber alongside their corresponding calculated results from the TPS.

**TABLE 2:** Results of gamma analysis at a gamma criterion of 3%/3 mm between experimental measurements from the MatriXX detectors and reference 2D proton dose distributions exported from the TPS.

	MatriXX		PPC05		
Plan type	10% Threshold	50% Threshold	Plan (cGy)	Measured (cGy)	Percentage difference (%)
Low‐dose plan	82.3%	98.8%	43.0	43.95	2.21
High‐dose plan	83.9%	98.9%	724.5	739.78	2.11

*Note*: Gamma analysis was performed using the myQA Patients software which is currently being used for our institution's IMPT patient‐specific QA measurement and analysis. Also shown are results of the absolute proton dose measurements using the PPC05 alongside with the results from the TPS calculations. For our institution's QA policy, a 3%/3 mm passing rate of 90% and an absolute proton dose agreement within 3% are required prior to treatment.

Abbreviation: TPS, treatment planning system.

### 3.3 Radiochromic film measurements and DVH calculations

**FIGURE 7:**
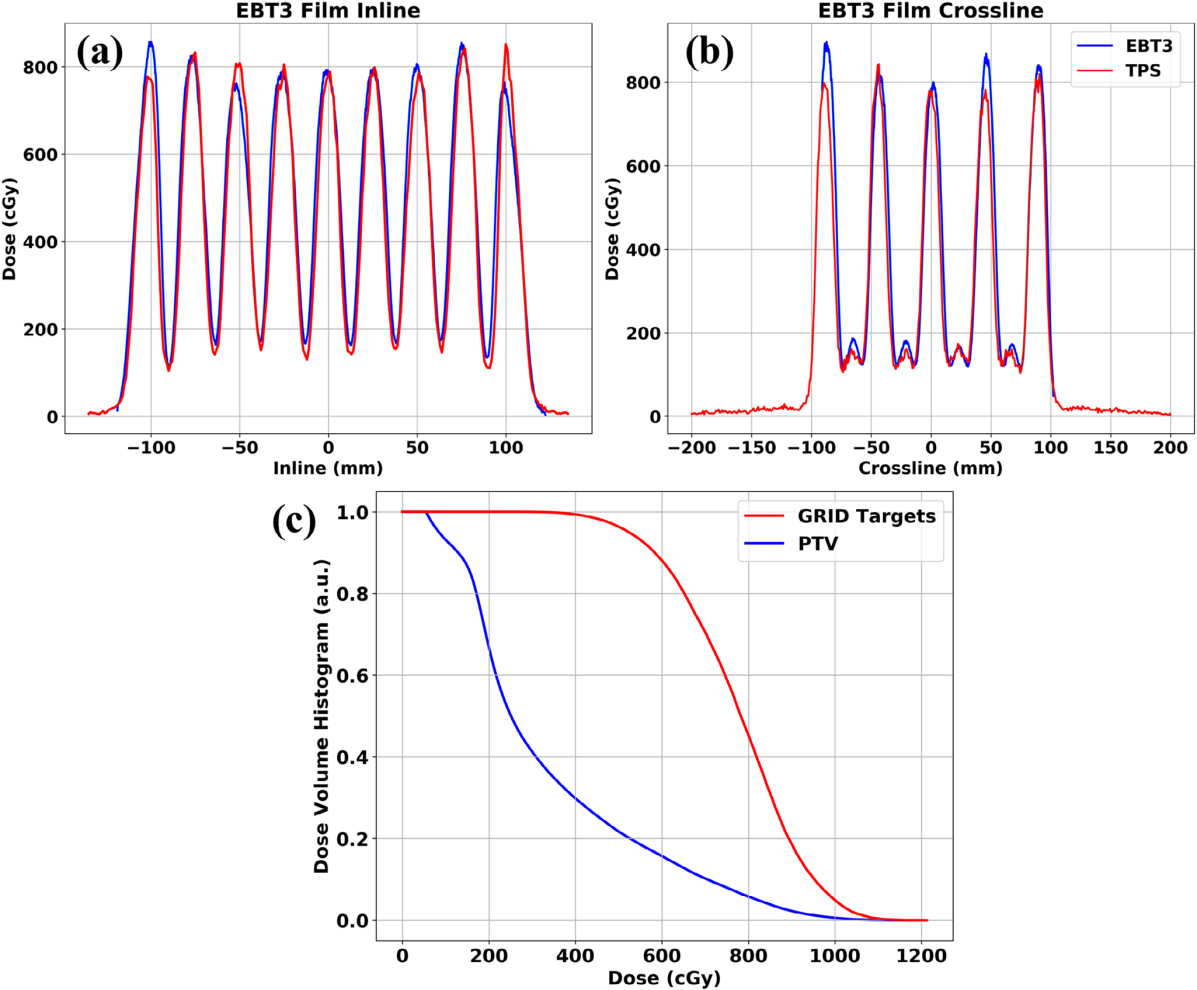
Results of the EBT3 radiochromic film absolute dosimetry of the high‐dose verification plan showing the 1D dose profiles along the (a) crossline and (b) inline directions. The small dose peaks within the crossline profiles are due to the merging of the adjacent nearest‐neighbor peaks within the inline direction (Figure 6). This effect is accounted for within our TPS calculations. The achievable PVDR was approximately 5.8. (c): DVH plots of the GRID high‐dose sub‐volume (red contour) within the bulky phantom PTV (blue contour). EUDs were determined to be 7.71 Gy for the GRID targets and 4.28 Gy for the larger PTV. PVDR, peak‐to‐valley‐dose‐ratio. EUD, equivalent‐uniform‐dose; TPS, treatment planning system.

Figure 7 shows the 1D absolute proton dose profiles along the (a) crossline and (b) inline directions through the central axis perpendicular to the proton beam's direction at isocenter. These 1D profile data were extracted from EBT3 radiochromic film measurements of the high‐dose verification plan. Profiles indicate agreement in the absolute proton dose magnitudes and positions of the peaks and valleys with their corresponding TPS values. Figure 7c contains the DVH plots of both the approximate phantom PTV and the cylinder sub‐volumes within the PTV. Table 3 reports the recommended spatially‐fractionated‐radiotherapy (SFRT) metrics by Zhang et al.^16^ The PVDR was measured to be approximately 5.8 and was consistent with TPS calculations.

**TABLE 3:** SFRT metrics dose reporting for the GRID phantom plan as generated for the high‐dose (8 Gy) prescription with 10 mm diameter optimization cylinders.

Contours	*D*_90_	*D*_50_	*D*_20_	*D*_10_	*D*_5_	*D*_mean_	PVDR	*D*_90_/*D*_10_	EUD
GRID subvolume	5.83	7.82	8.93	9.52	9.99	7.73	5.8	0.61	7.71
Scoring PTV	1.38	2.54	5.31	7.08	8.22	3.61		0.23	4.28

*Note*: All units are in Gy except for the PVDR and *D*_90_/*D*_10_ ratios which are dimensionless. Metrics were scored for GRID subvolume (red contour of Figure 4) and PTV scoring volume (cyan contour of Figure 4).

Abbreviations: EUD, equivalent‐uniform‐dose; SFRT, spatially fractionated radiation therapy.

### 3.4 Effect of simulated positional deviations of brass aperture

**FIGURE 8:**
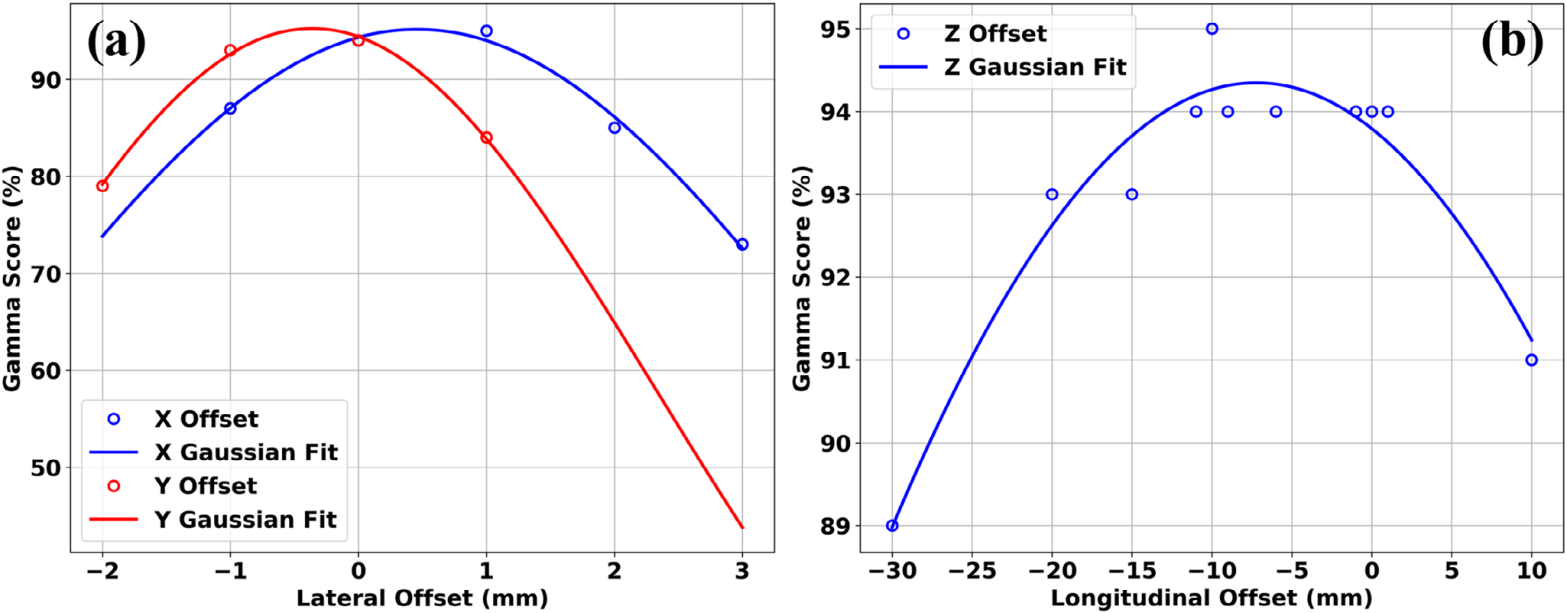
Plot of the calculated gamma scores (3%/3 mm gamma criteria) comparing the 2D experimental results (Lynx detector, low dose verification, 10 mm optimization cylinder diameter) and their corresponding 2D TPS calculations with simulated (a): lateral and (b): longitudinal position deviations of the brass aperture within the TPS. Gaussian curve fits were performed fitting the calculated gamma scores as a function of positional offsets. For lateral offsets along the x and y directions, the corresponding fits are $G_{x}\;\left(x\right)=95.2\%\times \exp\left(-{\left(x(\mathrm{mm})-0.459\;\mathrm{mm}\right)}^{2}/\left(2\times 3.45\;mm^{2}\right)\right)$ and $G_{y}\;\left(y\right)=95.2\%\times \exp\left(-{\left(y(\mathrm{mm})+0.360\;\mathrm{mm}\right)}^{2}/\left(2\times 2.70\;mm^{2}\right)\right)$ respectively. For longitudinal offsets along the z direction, the Gaussian fit is $G_{z}\;\left(z\right)=94.4\%\times \exp\left(-{\left(z(\mathrm{mm})+7.22\;\mathrm{mm}\right)}^{2}/(2\times 66.5\;mm^{2}\right))$. TPS, treatment planning system.

Figure 8 shows the results of the calculated gamma scores (3%/3 mm gamma criteria) comparing the 2D experimental results (Lynx detector, low dose verification, 10 mm optimization cylinder diameter) and their corresponding 2D TPS calculations with simulated (a): lateral and (b): longitudinal position deviations of the brass aperture within the TPS. These 2D gamma analyses were performed with PyMedPhys. Results showed a rapid decrease of the calculated 2D gamma scores with simulated positional offsets of the brass aperture in the lateral directions. However, the decrease of the calculated 2D gamma scores were more gradual for positional offsets of the brass aperture along the longitudinal direction. Gaussian fits were performed to empirically quantify this decrease of the calculated gamma scores with the magnitude of the positional offsets (Figure 8).

From the results of these Gaussian fits, the fitted *x*, *y*, and *z* positional offsets which corresponded to the maximum gamma scores were found to be 0.459, −0.360, and −7.22 mm respectively from nominal values. Their corresponding fitted 1σ values were found to be 3.45, 2.70, and 66.5 mm along the *x*, *y*, and *z* directions respectively. From these fitted 1σ values, the corresponding positional offset tolerances of the brass aperture positioning which maintained a gamma score of at least 90% at a gamma criterion of 3%/3 mm were calculated to be ± 0.818, ± 0.905, and ± 20.09 mm for the *x*, *y* and *z* directions respectively.

## 4 DISCUSSION

### 4.1 Considerations in brass collimator design

The brass collimator's manufactured hole diameter of 1.5 cm and center‐to‐center distances were chosen to replicate our institution's clinical experiences in delivering photon SFRT treatments for palliative intents within our institution. The hexagonal arrangement was chosen as it represented the most efficient high‐dose cylinder packing geometry which is also currently used within our clinical photon SFRT program. We note that there is a large degree of freedom available in setting the SFRT treatment parameters, that is diameters, center‐to‐center distances, PVDRs and so forth.^21^, ^40^ However, wherever possible, we have kept the most of these parameters similar to our photon experiences to compare the safety and efficacies of proton GRID SFRT treatments with our longstanding photon SFRT clinical experiences.

The brass collimator's thickness of 5 cm was determined to be sufficient in shielding the highest proton energies from our synchrocyclotron (227 MeV). In addition to these considerations, we have also incorporated the proton beam's divergence within the brass aperture's design. This inclusion was done to improve the deliverability, MU efficiency, and corresponding plan qualities of the collimated SFRT proton plans. Theoretically, if this divergence were not to be considered, the optimizer would force excess amounts of proton fluence through each aperture to confer adequate dose coverage to the SFRT targets, especially if they were to be geometrically mismatched with the natural beam divergence of the Mevion synchrocyclotron.

TPS calculations showed that the dose distributions at different depths are most influenced by the beam's divergence and, correspondingly, the brass aperture's divergent design. Shallower depths resulted in the decrease of the geometric center‐to‐center distances between dose peaks, whereas the opposite is true for deeper depths. The PVDR was found to be relatively consistent throughout the GRID PTV with this aperture design, with a slight increase at shallower depths. We did not find the center‐to‐center distances nor PVDR to be influenced by the proton energies or Bragg peak regions.

### 4.2 Gamma analyses

Gamma analyses as performed with both the manufacturer's provided QA software and PyMedPhys matched well, and thus for simplicity the latter was used for cases involving simulated positional deviations of the brass aperture's positioning (Figure 8). The best dosimetric agreements were found for verification plans that were optimized using optimization cylinder diameters of 10 mm. This result was seen for both the Lynx and the MatriXX detectors using both 2D gamma calculation algorithms. Using optimization cylinder diameters that were of the same physical size as the circular aperture increased the occurrence of proton spots being placed along the edges and body of the brass collimator which increased the magnitude of the dose calculation uncertainties due to increased proton scattering. These added dosimetric uncertainties due to edge‐scattered protons were previously noticed for patient‐specific collimators that were used for passively scattered proton beams and these effects were more apparent for small treatment fields.^41^, ^42^ As such, all subsequent analyses investigating the gamma scores for the high‐dose verification plan using the MatriXX detector and EBT3 radiochromic films, and subsequent investigations into the effects of the simulated positional deviations of the brass aperture were performed with optimization cylinder diameters of 10 mm.

Both low and high‐dose verification plans utilizing optimization cylinder diameters of 10 mm showed a gamma passing rate at 3%/3 mm gamma criteria that is well above our institution's QA policy requirement of 90% when calculated using both the actual manufacturer's software (Tables 1 and 2) and with independent PyMedPhys calculations (Figure 6). This has been validated for both the Lynx and MatriXX detectors using relative and absolute proton dosimetry, respectively over all 77 circular apertures (Figure 6). With these results, the physical brass aperture has been confidently commissioned for clinical use within our institution.

As mentioned earlier, accurate SFRT treatment planning utilizing the brass collimator is contingent upon the accurate digital geometrical and digital representation of the brass collimator within the TPS. The Monte Carlo engine considers the effects of attenuation and scattering of the proton beams with the brass collimator during treatment planning optimization. The high rates of passing of the Gamma analyses and close ion chamber agreements within these works indicate that the brass apertures have been sufficiently modeled. During the beam delivery, the proton pencil beam would be raster‐scanned across the entire face of the brass collimator with most of the beam fluence delivered through each aperture. The brass collimator improves the SFRT plan quality and increases the resultant SFRT's PVDR by decreasing the lateral penumbra and shielding the proton doses between each peak. Of the modelled processes, the scattering interactions was found to be more uncertain as seen in the relatively lower gamma score of the 15 mm optimization cylinder methods which sends an excess amount of proton fluence at the edges of the brass apertures thereby increasing the relative occurrence of scattering. This can be mitigated with the use of smaller diameter optimization cylinders.

### 4.3 Choice of detectors for routine patient‐specific QA

For initial testing purposes, the Lynx detector (Figure 3a) was extremely useful due to its sub‐millimeter spatial resolution (0.5 mm) and its reusability. However, there are two major disadvantages which limit it from effective routine clinical use: (a) the sensitive CCD of the Lynx detector tends to saturate easily and therefore makes it unfeasible for the measurement of therapeutic doses and (b) the optical signals that are collected by the CCD limits this detector to relative proton dosimetry only. From our results, we learned that an iris opening of 50% corresponded to a maximum deliverable dose of 50 cGy beyond the CCD would saturate.

Unlike the Lynx, the MatriXX detector can be used for absolute 2D dosimetry. However, it suffers from a poor spatial resolution (∼8 mm) as evidenced in Figure 6 which prevents the absolute proton doses of both the valleys and peaks to be measured simultaneously. However, we discovered that the absolute doses from the peaks can be reliably measured if the MatriXX detector's central element were to be aligned at isocenter. In addition to this, a more meaningful 2D gamma analyses can be performed with the MatriXX if we used a relatively higher dose threshold of 50% which effectively limited the gamma analyses regions strictly to the dose peaks. However, prior to its routine clinical use, both the valley dose and the peak dose should be established using higher‐resolution dosimetry methods.

Radiochromic films offer the highest spatial resolution and a dynamic range that is sufficiently large to measure both the peak and valley dose simultaneously (Figure 7). However, their non‐reusability, inter‐batch sensitivity variations, readout scanner positional and orientation dependence,^43^ and LET dependence^43^, ^44^, ^45^, ^46^, ^47^, ^48^ are serious limitations rendering them non‐ideal and tedious for routine patient‐specific QA measurement purposes.^43^, ^49^, ^50^ In addition to these uncertainties, it is also reported that radiochromic films will experience an under‐response in high linear‐energy‐transfer (LET) proton environments.^43^, ^44^, ^45^, ^46^, ^47^, ^48^ The LET dependence manifests as reduced dose responses, especially if the films were originally calibrated using TRS‐398 radiation environments at relatively shallower regions^37^ with relatively lower LET; there will be increased systematic uncertainties at intermediate to deeper depth measurements where higher LET values are prevalent. Under‐response of these radiochromic films of as high as 50% have been reported^51^ in literature which limits their recommended clinical use to profile measurements at relatively shallower depths away from the ends of the proton beams. These cumulative effects typically result in 1σ dosimetric uncertainties that are associated with absolute film dosimetry ranging from 2.6% to 4.1%,^49^ depending on the actual calibration methodologies undertaken.^50^

### 4.4 Positional errors

From Figure 8, it is demonstrated that the dosimetric accuracy of proton GRID treatments utilizing the brass aperture is contingent on its accurate positioning. This is especially so for deviations along the plane perpendicular to the proton beam direction where the positional tolerances are ± 1.5 mm beyond which there would be a steep falloff of the gamma pass rates due to the positional misalignment of the apertures. As for deviations along the beam direction, there is a greater tolerance of approximately ± 3 cm. Dose deviations that result from changes in the snout position will be caused by geometrical differences of the proton beam's divergence which are subtler than those deviations caused by offsets perpendicular to the beam direction.

Also from Figure 8, we have experimentally verified the positional agreements of the brass aperture between its digital representation within the TPS and its actual physical location when mounted on the gantry's snout to be within ± 1 mm. While not encountered in this study, any such disagreements can be easily rectified iteratively by (a) obtaining the experimental 2D dose distribution using a Lynx detector using a low‐dose plan, (b) performing a gamma analysis, (c) determining the positional shifts required to maximize the gamma analysis, (d) correcting the digital position of the brass aperture within the TPS systematically with global shifts, and (e) repeating the entire measurement and gamma analysis process again. However, this iterative correction process is contingent on the ability to position the Lynx QA device accurately with its central pixel coincident with the beam's isocenter. This may be set‐up with the help of gantry's x‐ray imaging system or external lasers but their respective isocenter agreements should be verified to be within ± 1 mm prior to their use. If there is an uncorrected systematic deviation between the setup of the Lynx QA device with the beam's isocenter necessitating a global shift, this iterative digital correction process or the positional verification of the brass aperture will not be possible.

### 4.5 Creation of phantom for routine patient‐specific QA

Patient‐specific QA of the proton GRID plans should be performed prior to beam delivery.^52^ This should involve a recalculation of the clinical plan utilizing the brass apertures on a digital water phantom with the 2D planar dose at the measurement plane exported for gamma analysis. To achieve this, we created a digital phantom within “RayPhysics” which contained a brass aperture that was centered at a fixed snout extension of 19.5 cm. However, when approved within RayPhysics and subsequently used for patient‐specific QA recalculation within RayStation, the brass aperture's position will be fixed relative to the surface of the “External” contour of the digital phantom. This can create problems for a proton patient‐specific QA program where 2D planar measurements are performed isocentrically as any shifts in the isocenter of the digital phantom during the QA plan creation process will induce an undesired shift of the brass aperture relative to the gantry's snout. There are two potential solutions to remedy this issue: (a) a constant depth of measurement be performed for isocentric setups, or (b) non‐isocentric measurements be performed for patient‐specific QA with the brass aperture. The former solution is impractical due to the large variability of patient anatomies and tumor geometries. For the former scenario, a new digital QA phantom must be created within RayPhysics every time a new isocentric measurement depth is desired. Thus, we recommend the use of the latter solution which will utilize a fixed setup of the clinical water phantom relative to the brass aperture and off‐isocenter 2D planar measurements if different depth measurements are required for patient‐specific QA. For patient‐specific QA, we recommend the choice of a depth which reasonably intersects all high‐dose cylinders of the GRID plan.

### 4.6 Recommended physics tests prior to clinical use

Prior to clinical use, we recommend assessment of the digital representation of the brass aperture as support structures within the TPS. This includes verification of the agreement between the brass aperture's digital positioning within the TPS and its actual physical placement when mounted upon the gantry's snout, and also an assessment of the accuracy of the digital modeling of the aperture's divergence relative to the proton beam. This should be verified using a 2D detector of a high‐spatial resolution, with appropriate options including radiochromic films or the Lynx scintillation detector. For the Lynx detector, the delivered MUs and iris settings should be carefully selected to avoid detector saturation. Any systematic positional discrepancies can be corrected for iteratively (Section 4.3). This test should be performed across all of the divergent apertures that are milled within the brass collimator. A gamma analysis using the clinical passing criteria should be performed.

Once this is done, a dosimetric verification should be performed. This can be undertaken using absolute film dosimetry, an ionization chamber array such as the MatriXX detector or a single point measurement at depth using an ionization chamber. For ion chamber point dose measurements, the active ionization chamber volume should be contoured with the average dose reported to account for partial volume averaging effects.

After above tests were done and proven acceptable, a clinical procedure for patient‐specific QA should be developed. We recommend creating a digital QA phantom and the use of fixed setup, non‐isocentric measurement techniques for expedient measurements (Section 4.4). Once the brass collimator has been comprehensively commissioned, patient‐specific QA may involve a 2D MatriXX or film measurement within a relevant depth that assesses for all proton doses to be delivered across all of the used apertures.

### 4.7 Future studies

Future developments will include the clinical utilization of the brass apertures to generate proton GRID plans. Dosimetric comparisons between multiple techniques such as a single‐gantry traditional GRID technique of a cross‐fired GRID approach will be performed for a variety of treatment sites.

Within these works, we have generally used the aforementioned collimator design to create proton SFRT plans that are closely matching with their counterparts from our longstanding photon SFRT clinical program. As mentioned earlier, the magnitude of the PVDR was determined to be relatively constant within the approximate therapeutic depths of 5–15 cm which represent the vast bulk of our patients. Future works will involve the fabrication of patient‐specific brass collimator designs as a function of their mean therapeutic depth to confer a more constant center‐to‐center peak dose distances over a wider depth range. Commissioning will involve the repeat of the methods as described within these works for each collimator design.

## 5 CONCLUSION

We have successfully designed, fabricated, and experimentally tested a brass GRID collimator containing divergent‐matched apertures to support proton SFRT treatments on a gantry‐mounted proton therapy system. Our results indicated that a potential deployment of this SFRT technique would need relative and absolute 2D dose measurements using a combination of 2D detectors, and a 1D absolute dose measurement using an ion chamber within a water phantom, and a verification with the TPS calculations as well.

## CONFLICT OF INTEREST STATEMENT

The authors declare no conflicts of interest.
